# Characterization of intravascular cellular activation in relationship to subclinical atherosclerosis in postmenopausal women

**DOI:** 10.1371/journal.pone.0183159

**Published:** 2017-09-14

**Authors:** Muthuvel Jayachandran, Vesna D. Garovic, Michelle M. Mielke, Kent R. Bailey, Brian D. Lahr, Virginia M. Miller

**Affiliations:** 1 Departments of Surgery, Mayo Clinic, Rochester, Minnesota, United States of America; 2 Physiology and Biomedical Engineering, Mayo Clinic, Rochester, Minnesota, United States of America; 3 General Internal Medicine, Division of Nephrology and Hypertension, Mayo Clinic, Rochester, Minnesota, United States of America; 4 Health Science Research, Division of Epidemiology, Mayo Clinic, Rochester, Minnesota, United States of America; 5 Neurology, Mayo Clinic, Rochester, Minnesota, United States of America; 6 Health Science Research, Division of Biostatistics, Mayo Clinic, Rochester, Minnesota, United States of America; Maastricht University, NETHERLANDS

## Abstract

**Objective:**

Mechanisms and interactions among intravascular cells contributing to development of subclinical atherosclerosis are poorly understood. In women, both menopausal status and pregnancy history influence progression of atherosclerosis. This study examined activation and interactions among blood elements with subclinical atherosclerosis in menopausal women with known pregnancy histories.

**Methods:**

Carotid intima-media thickness (CIMT), as a marker of subclinical atherosclerosis, was measured using B-mode ultrasound in age- and parity-matched women [40 with and 40 without a history of preeclampsia] 35 years after the index pregnancy. Interactions among intravascular cells (38 parameters) were measured by flow cytometry in venous blood. Data analysis was by principal component which retained 7 independent dimensions accounting for 63% of the variability among 38 parameters.

**Results:**

CIMT was significantly greater in women with a history of preeclampsia (P = 0.004). Platelet aggregation and platelet interactions with granulocytes and monocytes positively associated with CIMT in postmenopausal women independent of their pregnancy history (ρ = 0.258, P< 0.05). However, the association of the number of platelets, platelet activation and monocyte-platelet interactions with CIMT differed significantly depending upon pregnancy history (test for interaction, P<0.001).

**Conclusion:**

Interactions among actived intravascular cells and their association with subclinical atherosclerosis differ in women depending upon their pregnancy histories.

## Introduction

Conventional risk factors for cardiovascular disease such as age, blood pressure, dyslipidemia and smoking status do not accurately reflect future cardiovascular risk for women [1, 2]. Pregnancy related complications such as preeclampsia (PE) increase risk for future hypertension, ischemic heart disease, stroke, and premature cardiovascular death in women [3]. However, mechanisms and factors contributing to the increased risk are poorly understood but may reflect a constellation of cardio-metabolic parameters that may exist prior to pregnancy or are exacerbated during the pregnancy and persist thereafter [4].

Activation of platelets and leukocytes contribute to progression of atherosclerosis [5]. In addition, cells activated by oxidative stress, cytokines, antigens or receptor ligands shed double membrane bound extracellular microvesicles (MV, also known as microparticles) that differ from mineral complexes, lipids or protein particles of similar size (0.04μm-1μm). MV are involved in the transfer of bioactive molecules such as RNAs, proteins, receptors, and metabolites from the parent cells of origin to other cells initiating signaling events that can contribute to pathological processes [6–9]. The number and characteristics (expressions of surface proteins and receptors and the content of bioactive molecules) of MV differ depending on their cellular origins and the processes that initiate their formation.

During pregnancy, activation of intravascular cells (platelets, leukocytes, and lymphocytes) are greater in women with preeclampsia (PE) compared to matched women with normotensive pregnancies (NP) [10–12]. A history of PE may sustain a pro-thrombotic and pro-inflammatory state which contributes to the accelerated development of cardiovascular disease. However, intravascular cellular activation and their interactions with specific populations of blood-borne MV in menopausal women with histories of PE have not been examined. Therefore, the present study was designed to provide an extensive characterize intravascular cellular activation in association with a measure of preclinical atherosclerosis in postmenopausal women in whom pregnancy histories were known. Two hypotheses were tested: 1) specific types of activated intravascular cells, blood-borne MV, and their interactions would associate with a measure of subclinical atherosclerosis (carotid intima-media thickness, CIMT); and 2) these would differ between postmenopausal women depending upon their pregnancy histories.

## Methods

### Study design and participants

This study was approved by the Institutional Review Boards at Mayo Clinic and Olmsted County Hospital, Rochester, MN. All participants gave written informed consent. The Rochester Epidemiology Project medical records-linkage system was used to identify women (n = 40) with histories of PE and age- and parity-matched women (n = 40) with histories of NP who gave birth from 1976 through 1982 [13]. Because the purpose of the study was to examine processes contributing to development of subclinical atherosclerosis, women with medical-record confirmed clinical diagnoses of the following conditions were excluded: myocardial infarction, congestive heart failure, stroke, dementia, any cancer (with the exception of non-melanoma skin cancer), autoimmune disease (e.g., multiple sclerosis, lupus), and neurological conditions (e.g., epilepsy).

### Measurement of CIMT

CIMT was measured using high-resolution B (brightness)-mode ultrasound, as described previously [14]. A single individual who was blinded to each woman’s pregnancy history read the images. Mean coefficient of variation of measures of CIMT on two scans from a single individual range from 0.0%-7.7% [15]. The measurement of CIMT is expressed as millimeters (mm).

### Blood collection

Blood was collected in the early morning after overnight fasting from antecubital venipuncture with a 21 gauge needle (with initial 2mL discarded) for the measurements of platelet reactivity, blood-borne MV, and intravascular cell-cell interactions. The anticoagulant used for each assay was dictated by the requirement of that assay as described previously [16, 17]. Samples were processed for each assay within 30 minutes of sample collection [18].

### Blood platelet reactivity assays

Blood platelets and mean platelet volume were measured by Beckman Coulter^®^ Ac.T diff 2 Hematology Analyzer counter, Division of Hematology Research, Mayo Clinic, Rochester, MN. *Whole blood platelet aggregation* was measured by lumi-aggregometer, Chrono-Log Corporation, Model 700, Havertown, PA. *Platelet dense granular ATP secretion* in diluted platelet rich plasma was measured in real time by bioluminescence using premixed firefly luciferase (0.5 mg/mL Hanks’ medium) and luciferin (5mM in Hanks’ medium) at 30°C and a final platelet concentration of 250–500 platelets/μL [16, 17]. Measurement of phosphatidylserine (annexin-V binding), P-selectin, and fibrinogen receptor (PAC-1 binding) on platelet surfaces under basal conditions was evaluated using standard flow cytometry [16, 17].

### Characterization of intravascular cell-cell interactions

Cell-cell interactions were identified using antibodies and methods previously validated and published by our group [19, 20] with slight modifications. In general, diluted (1:100 in H/H buffer pH 7.4) whole blood (100 μL) was incubated with 3 μL of fluorophores [either fluorescein (FITC) or phycoerythrin]-conjugated to cell surface specific antibodies as follows. After 30 minutes incubation, 1% paraformaldehyde (400 μL) was added to the mixture. Matched fluorophore -conjugated isotype antibodies were stained simultaneously as controls to set the threshold and exclude non-specific binding. All interactions among intravascular cells (platelets or leukocytes or vascular endothelium) with cell-derived MV and phosphatidylserine expression on activated platelets were analyzed by digital flow cytometry (FACSCanto^TM^, BD Biosciences, San Jose, CA).

*Antibodies used to determine interactions of platelets with leukocytes and vascular endothelium* were platelet (CD42a)—antibody in combination with antibodies for common leukocytes (CD45), granulocytes (CD15), monocytes (CD14), T-lymphocytes (CD3), B-lymphocytes (CD19), and vascular endothelium (CD62E) and / or with fluorophore conjugated recombinant annexin-V (binds to surface phosphatidylserine). Platelets labeled with fluorophore conjugated CD42a antibody were identified by forward and side scatter. Ten thousand gated events (counts) were collected for each sample. The number of platelets positive for antigens for leukocytes and endothelial cells are expressed as percentages of platelets positive from a total 10,000 gated platelet events.

*Antibodies used to determine interactions of leukocytes with platelets and vascular endothelium* were allophycocyanin (APC)—conjugated common leukocyte (CD45)—antibody in combination with phycoerythrin conjugated antibodies for platelets (CD42a), and vascular endothelium (CD62E), and / or with FITC conjugated annexin-V (binds to surface phosphatidylserine). Blood cells were counted using a Beckman Coulter^®^ Ac.T diff 2 Hematology Analyzer counter, Division of Hematology Research, Mayo Clinic, Rochester, MN. Leukocytes labeled with APC-conjugated CD45 antibody were identified by forward and side scatter; 5,000 gated leukocyte events were collected for each sample. The number of platelet- and endothelial- antigen positive granulocytes, monocytes, and lymphocytes are expressed as percentages of platelet- and / or endothelial- antigen positive granulocytes, monocytes, and lymphocytes from total granulocytes, monocytes, and lymphocytes of 5,000 gated CD 45 positive leukocyte events, respectively.

### Isolation, identification, and characterization of blood-borne MV

Detailed standardized methodologies of MV isolation from blood anticoagulated with protease inhibitors (1 μM hirudin plus 10 μM soybean trypsin inhibitor) by differential centrifugation, identification, and characterization by digital flow cytometry were as previously published [18, 20]. The concentration of blood-borne MV is expressed as MV/μL plasma.

### Statistical analyses

Demographic, clinical and MV data were described with quartiles (median [50^th^ percentile], lower quartile [25^**th**^ percentile], and upper quartile [75^th^ percentile]), or with absolute numbers and percentages. Because the 38 variables are likely correlated and contain redundancies (i.e., cells positive for more than one cell-specific antigen), principle components (PC) analysis was performed to reduce these data to a smaller number of independent factors by identifying variable clusters and summarizing each into a single score. As most of the 38 continuous variables were skewed, each was transformed into rank-based, normalized measures, specifically using the probit transformation. Once patterns among these inputs were explored and translated into PCs, the association of preeclampsia with each PC was analyzed using a t-test. Next, to assess the relationships of blood elements and MV with the measure of subclinical atherosclerosis, each PC was correlated with CIMT measurements using a nonparametric Spearman correlation coefficient. To account for the sampling frame, correlations were computed separately for the groups with prior histories of NP and PE, and partial correlations were obtained on the pooled set of women adjusting for group status. In addition, a multivariable proportional odds model was constructed that included CIMT as the dependent variable and each PC term and PE group indicator as main effects, along with their cross-product (interaction) terms, and screened for a differential relationship between PCs and CIMT according to PE status using a global test for interaction. This analysis was done by simultaneously testing the group of interactions for significance (i.e., a multiple degree-of-freedom likelihood ratio test), with additional testing of the individual interaction terms only if the global test was significant.

## Results

### General characteristics

All but one participant was white Caucasian and all were matched for both their current ages and ages at index pregnancy. At the time of the study, approximately 35 years after the index pregnancy, body mass index, waist circumference, circulating levels of insulin, and high-sensitive C-reactive protein were higher in women with histories of PE compared to those with histories of NP (**Table 1**). Women with histories of PE were more frequently taking anti-hypertensive medications. CIMT was significantly greater in women histories of PE (**Table 1**).

**Table 1:** Characteristics of women with a history of normotensive and preeclamptic pregnancy.

*Variable*	*History of normotensive pregnancy* *(n = 40)*	*History of preeclamptic pregnancy* *(n = 40)*	*P-value*
Age at study consent	59.6 (56.2,62.5)	59.2 (56.3,62.5)	0.814
Age at 1^st^ live birth	24.0 (22.3, 26.3)	24.5 (21.7, 25.8)	0.913
Antihypertensive meds, chart-abstracted	**5 (13%)**	**23 (28%)**	**<0.001**
Lipid statin meds, self-reported	5 (13%)	9 (23%)	0.239
Aspirin, self-reported	6 (15%)	11 (28%)	0.172
Anti-inflammatory meds, self-reported	29 (50%)	27 (68%)	0.112
Past or current hormone therapy	17 (43%)	17 (43%)	1.000
**Clinical parameters**			
Body mass index (kg/m^2^)	**25.3 (23.1, 32.0)**	**29.8 (25.9, 33.7)**	**0.023**
Waist circumference (cm)	**85.3 (79.3, 99.6)**	**98.0 (88.3, 104.0)**	**0.009**
Systolic blood pressure (mm Hg)	128.7 (116.5, 145.7)	131.7 (119.7, 140.2)	0.613
Diastolic blood pressure (mm Hg)	75.2 (69.7, 84.0)	79.7 (69.3, 83.3)	0.368
Hypertension, chart-abstracted (n)	**8 (20%)**	**24 (60%)**	**<0.001**
Mean CIMT (mm)	**0.73 (0.70, 0.78)**	**0.80 (0.75, 0.85)**	**0.004**
**Blood chemistry**			
Total cholesterol (mg/dL)	204.5 (182.0, 222.5)	189.5 (168.0, 215.0)	0.095
LDL Cholesterol (mg/dL)	123.0 (99.7, 136.4)	106.1 (87.9, 124.3)	0.087
HDL Cholesterol (mg/dL)	64.0 (50.5, 76.5)	54.5 (41.0, 69.5)	0.054
Triglycerides (mg,dL)	97.5 (72.0, 123.5)	108.0 (85.0, 163.0)	0.078
Fasting glucose (mg/dL)	95/5, (91.0, 101.5)	98.0 (91.5, 109.5)	0.151
Insuin (μlU/mL)	**4.6 (3.3, 6.0)**	**7.1 (4.7, 14.8)**	**<0.001**
Tumor necrosis factor alpha (pg/mL)	1.3 (1.0, 2.6)	1.3 (0.9, 1.4)	0.580
Interleukin-6 (pg/mL)	1.6 (1.0, 2.6)	1.9 (1.2, 3.8)	0.243
hs-CRP (mg/dL)	**0.10 (0.06, 0.17)**	**0.19 (0.12, 0.29)**	**0.002**

Note: Continuous variables are reported as median (IQR); n (%); groups were compared using Wilcoxon rand sum test; Abbreviations: CIMT, carotid intima-media thidknwss; HDL, high density lipoprotein; hs-CRP, high sensitivity C-reactive protein; LDL, low density lipoprotein.

### Activated cellular elements of the vascular compartment

Measures of the 38 parameters of cell counts, cellular activation, MV and cell-cell interactions are provided in **Table 2**. PC analysis yielded 7 independent dimensions that accounted for 63% of the variability among the 38 variables. The values of the highest factor loadings are presented for each PC in **Table 3**.

**Table 2:** Measures of blood cells, platelet characteristics, populations of microvesicles and cell-cell interactions in women with a history of normotensive or preeclamptic pregnancy.

*Variable*	*History of Normotensive* *Pregnancy* *(n = 40)*	*History of Preeclamptic Pregnancy* *(n = 40)*
**Blood cells**		
Platelet count (10^3^/μL)	275.0 (226.5, 313.5)	279.0 (249.0, 311.5)
White blood cell count (10^3^/μL)	4.9 (4.2, 5.9)	5.3 (4.8, 6.2)
Granulocyte count (10^3^/μL)	3.1 (2.7, 3.9)	3.7 (3.2, 4.3)
Lymphocyte count (10^3^/μL)	1.5 (1.2, 1.6)	1.5 (1.3, 1.8)
Monocyte count (10^3^/μL)	0.3 (0.3, 0.4)	0.3 (0.3, 0.4)
**Platelet characteristics**		
Mean platelet volume (fL)	8.0 (7.5, 8.4)	7.8 (7.2, 8.2)
Whole blood platelet aggregation (amplitude)	21.0 (18.5, 24.0)	21.0 (19.5, 24.0)
ATP Secretion (amoles/platelets)	14.3 (10.8, 16.7)	13.6 (10.8, 16.0)
Basal expression of phosphatidylserine (annexin V) (%)	6.0 (4.5, 8.9)	4.4 (3.4, 6.5)
Basal expression of P-selectin (%)	1.2 (0.6, 2.1)	1.4 (0.8, 1.9)
Basal expression of fibrinogen receptor (PAC-1,%)	0.7 (0.4, 1.5)	0.6 (0.4, 1.3)
**Microvesicles (MV/ μL)**		
Platelet-derived (CD42a)	831.6 (480.0, 1224.1)	679.0 (557.8, 1208.1)
Leukocyte-derived (CD45)	14.4 (10.3, 16.9)	14.6 (10.1, 22.2)
Erythrocyte-derived (CD235a)	27.5 (20.4, 41.7)	32.4 (19.3, 46.6)
Endothelium-derived (CD62E)	6.5 (4.4, 9.9)	5.6 (3.9, 8.4)
Smooth muscle cell-derived (SM22α)	1.0 (0.7, 2.1)	1.5 (0.7, 2.9)
Stem/Progenitor cell (CD117)	4.1 (3.2, 6.1)	7.3 (3.9, 11.0)
Adipocyte-derived (Pref-1)	7.8 (4.8, 9.6)	8.7 (4.9, 15.3)
Senescent cell-derived (P16-set)	0.9 (0.5, 1.5)	1.0 (0.6, 2.1)
Inter cellular adhesion molecule-1 (ICAM-1)	3.6 (2.8, 6.0)	3.5 (2.6, 5.7)
Vascular cell adhesion molecule-1 (VCAM-1)	1.5 (1.0, 1.9)	1.9 (0.9, 2.6)
Phosphatidylserine (annexin-V)	1018.9 (651.9, 1241.8)	865.2 (651.1, 1450.3)
**Tissue factor (TF)**	10.8 (7.2, 16.8)	15.7 (9.0, 31.1)
**Interactions among cellular elements (%)**		
Platelets pos. for leukocyte (CD45) antigen	3.2 (2.7, 4.3)	2.8 (2.2, 3.7)
Platelets pos. for granulocyte (CD15) antigen	2.7 (2.0, 3.1)	2.0 (1.3, 2.7)
Platelets pos. for monocyte (CD14) antigen	3.6 (2.3, 4.6)	2.8 (2.0, 3.8)
Platelets pos. for T-lymphocyte (CD3) antigen	1.7 (1.5, 2.3)	1.7 (1.5, 2.2)
Platelets pos. for B-lymphocyte (CD19) antigen	1.5 (1.2, 1.8)	1.7 (1.3, 2.2)
Platelets pos. for endothelial (CD62E) antigen	4.1 (2.6, 6.2)	3.4 (2.3, 4.1)
Granulocytes pos. for annexin-V	21.1 (9.2, 42.1)	25.5 (11.7, 36.1)
Granulocytes pos. for platelet (CD42a)antigen	18.3 (14.9, 24.2)	15.7 (11.9, 20.2)
Granulocytes pos. for endothelial (CD62E) antigen	24.0 (8.4, 41.1)	23.7 (12.3, 38.7)
Monocytes pos. for annexin-V	21.4 (10.5, 30.0)	20.3 (10.6, 29.4)
Monocytes pos. for platelet (CD42a) antigen	20.4 (16.1, 30.5)	19.9 (14.1, 28.6)
Monocytes pos. for CD62E	27.1 (14.7, 41.2)	25.0 (14.8, 35.3)
Lymphocytes pos. for annexin-V	2.9 (2.6, 4.1)	2.8 (1.8, 3.8)
Lymphocytes pos. for platelet (CD42a)antigen	3.0 (2.2, 3.9)	2.3 (1.7, 4.3)
Lymphocytes pos. for endothelial (CD62E) antigen	12.0 (9.5, 16.9)	9.7 (2.1, 17.7)

Abbreviations: ATP, adenosine triphosphate; pos., positive

**Table 3:** Loadings of individual cell, microvesicles and cell-cell interaction parameters on principal components.

*Measure Loadings*:	*PC#1*	*PC#2*	*PC#3*	*PC#4*	*PC#5*	*PC#6*	*PC#7*
**Blood cells**							
Blood platelet count						0.41	
Total white blood cell count			0.43				
Granulocyte count			0.38				
Lymphocyte count			0.35				
Monocyte count							
**Platelet characteristics**							
Mean platelet volume						-0.49	
Whole blood platelet aggregation				0.34			
ATP Secretion					0.44	-0.24	
Basal expression of phosphatidylserine (annexin-V)	0.25					-0.22	
Basal expression of P-selectin					0.28		0.29
Basal expression of fibrinogen receptor (PAC-1)					0.25		
**Blood-borne microvesicles**							
Platelet-derived (CD42a)			-0.33	0.24			
Leukocyte-derived (CD45)		0.22			0.23		
Erythrocyte-derived (CD235a)			-0.25				
Endothelium-derived (CD62E)		0.34					
Smooth muscle cell-derived (SM22α)		0.25					
Stem/progenitor cell (CD117)				-0.23			
Adipocyte-derived (Pref-1)		0.31					
Senescent cell-derived (P16-set)		0.33					
Inter cellular adhesion molecule-1 (ICAM-1)		0.29					
Vascular cell adhesion molecule-1 (VCAM-1)		0.33					
Phosphatidylserine (annexin-V)			-0.35	0.21			
Tissue factor		0.27		-0.23			
**Interactions among cellular elements**							
Platelets pos. for leukocyte (CD45)							
Platelets pos. for granulocyte (CD15)					0.25		-0.22
Platelets pos. for monocyte (CD14)	0.27						
Platelets pos. for T-lymphocyte (CD3)					0.26		-0.49
Platelets pos. for B-lymphocyte (CD19)					0.29		-0.42
Platelets pos. for endothelial (CD62E)	0.29						
Granulocytes pos. for phosphatidylserine (annexin-V)	0.28			-0.22			0.30
Granulocytes pos. for platelets (CD42a)				0.29			0.23
Granulocytes pos. for endothelium (CD62E)	0.28			-0.22			0.27
Monocytes pos. for phosphatidylserine (annexin-V)	0.30						0.20
Monocytes pos. for platelets (CD42a)				0.31	0.27	0.31	
Monocytes pos. for endothelium (CD62E)	0.31						
Lymphocytes pos. for phosphatidylserine (annexin-V)							
Lymphocytes pos. for platelets (CD42a)				0.21			
Lymphocytes pos. for endothelium (CD62E)	0.22						
*Proportion*	*19*.*1%*	*12*.*7%*	*8*.*6%*	*7*.*2%*	*6*.*0%*	*5*.*1%*	*4*.*6%*
*Cumulative*	*19*.*1%*	*31*.*8%*	*40*.*4%*	*47*.*6%*	*53*.*6%*	*58*.*7%*	*63*.*3%*

In a multivariable logistic model with the dependent variable indicating pregnancy group membership, the 7 PCs were analyzed simultaneously as a set of predictor variables and tested for any group differences. This global test showed no overall association between the PCs and history of PE (P = 0.146, 7 d.f.). However, PC#1, largely a function of basal activation of platelets, granulocytes and monocytes (expression of annexin V), and the interrelationships among circulating blood cells with each other and the endothelium, accounted for 19% of the total variance, and showed a nominal difference between groups (P = 0.045, 1 d.f.).

### Cellular activation and associations with subclinical atherosclerosis

Independent of pregnancy history, the global test for assessing the association between PCs and CIMT was not significant (P = 0.364; **Table 4**). However, within the NP group, the global test, in which all 7 PCs were assessed simultaneously, showed a significant association with CIMT (P = 0.009, 7 d.f.). Individually significant correlates of CIMT in the NP group included measures of whole blood platelet aggregation, activation of platelets, and reflects interactions among monocytes and granulocytes with vascular endothelium and number of microvesicles positive for tissue factor (PC#4; Spearman’s ρ = 0.327; P = 0.039), and measures of platelet count, platelet activation and interrelationships of monocytes with platelets (PC#6; ρ = -0.359; P = 0.022). Within the PE group, the global test involving the combined set of PC predictors was insignificant (P = 0.481, 7 d.f.), reflecting no individual associations with CIMT.

**Table 4:** Association of specific principal components with CIMT by pregnancy history.

Analysis Performed on PCs	*GlobalTest* ^‡^	PC#1	PC#2	PC#3	PC#4	PC#5	PC#6	PC#7
Correlation with CIMT				Spearman ρ^+^				
Group with history of normotensive pregnancy	*P = 0*.*009*	-0.130	-0.055	-0.037	0.327*	-0.169	-0.359*	0.181
Group with history of preeclampsia	*P = 0*.*481*	0.275	-0.139	0.120	0.117	0.030	0.219	-0.142
Both groups pooled together	*P = 0*.*364*	0.050	-0.077	0.012	0.258*	-0.068	-0.014	0.035
Test of interaction	*P = 0*.*014*						***	

^+^ Group-specific results are reported as Spearman’s ρ rank correlation coefficients, which measure the strength of association of each PC with carotid intima-media thickness (CIMT); group-combined results represent partial Spearman ρ values which measure each correlation controlling for the effects of PE group. Tests for significant correlations are denoted with symbols as described below.

^*‡*^For each of the two groups and the combined group, a global test for any association among the 7 PCs with CIMT was performed in a multivariable ordinal logistic model with CIMT as the dependent variable and the PCs simultaneously entered as predictor variables (i.e., 6 degree-of-freedom likelihood ratio test comparing this model to a reduced model without PC predictor terms). As a global test for interaction, a model was constructed that additionally included a PE group indicator and the cross-product (interaction) terms between PE indicator and each PC, with the group of interactions tested for significance by formally comparing these two models with and without interaction terms (6 degree-of-freedom likelihood ratio test)

* *P* < 0.05

*** *P* < 0.001.

Several of the individual correlations of the PC with CIMT differed both in direction (positive or negative) and magnitude between pregnancy groups. In a multivariable model that included each PC and a PE group indicator as main effects as well as their cross-product (interaction) terms, the overall test for the presence of one or more interactions was significant (P = 0.014, 7 d.f.; **Table 4**). Specifically, the interaction term corresponding to platelet count, measures of platelet activation, and interrelationships of monocytes with platelets antigen (PC#6) showed a difference in the correlation with CIMT between PE (ρ = 0.219) and NP (ρ = -0.359) groups (test of individual interaction, P<0.001, 1 d.f.).

## Discussion

Results of the present study provide evidence that pregnancy history, in particular, a history of preeclampsia, influences cellular activation and their interactions that associate with a measure of subclinical atherosclerosis up to 35 years after the index pregnancy. These results are unique in that they provide a broad assessment of intravascular cellular activation in postmenopausal women who had no prior diagnosis of myocardial infarction, angina, heart failure, stroke or cerebral vascular disease.

Previous analysis of this cohort confirmed that PE was predictor of coronary arterial calcification [13]. Number of circulating tissue factor, intercellular adhesion molecule-1, stem cells, and adipocytes antigen positive blood-borne MV associated with coronary artery calcification in postmenopausal women with histories of PE [21]. Results of the present study extend these observations to address the influence of pregnancy history on the relationship of cellular activation with a measure of subclinical atherosclerosis, i.e. CIMT (21). This study is consistent with some but not all observations that having a history of PE increases the trajectory for increases in CIMT[22, 23] and provides new evidence that pregnancy history influences intravascular celllular activation including cell-derived blood-borne MV that may influence progression of CIMT. A significant association between 7 PC of vascular activation and CIMT was observed only in the NP group. Differences in directionality (negative or positive association) of the PCs with CIMT by pregnancy history may reflect either processes of cellular activation that might reflect differences of increased body mass index, hypertension, and insulin sensitivity or the depletion of activated cells from the circulation due to adherence to other blood-borne cells or the vascular endothelium, or participation in micro-thrombosis in the capillaries. This latter possibility of depletion of the cells from the circulation is supported by the global difference between groups of PCs that reflects basal levels of platelet activation, and platelet, granulocyte, monocyte, and lymphocyte interactions with each other and the vascular endothelium (PC #1) and by the significant interaction of the PC that reflects platelet count, platelet activation and their interactions with monocytes (PC#6). Blood platelets are a major regulator of normal hemostasis and thrombosis and are activated early in the development of vascular disease [16, 24, 25]. The interactions identified by these cellular interactions suggest that factors associated with a history of PE and ongoing conventional cardiovascular risk factors alter the profile of intravascular cellular activation that could initiate or accelarate the progression of subclinical atherosclerosis.

In addition to platelet activation, PE is also characterized by an exaggerated vascular (endothelium) inflammatory response, and leukocyte, granulocyte, and monocyte activation [10, 26–30].

Unknown factors resulting from the preeclampsia in combination with perhaps conventional cardiovascular risk factors (in particular, those related to hypertension, adiposity and insulin resistance) may sustain an exaggerated inflammatory response.

During physiological and pathological processes, MV shed from specific types of activated cells will reflect the micro-environment in which they were generated [6, 31, 32]. Platelet-derived MV are the most abundant type of MV in the circulation of healthy men and women [20, 33]. Numerous studies have identified changes in circulating MV from various cell types during preeclampsia [34–36]. Indeed, pro-thrombotic MV and those shed from activated vascular endothelium, and leukocytes associated with CIMT in healthy, recently menopausal women in whom pregnancy histories were unknown [17]. MV derived from monocytes, macrophages, vascular smooth muscle cells and those positive for tissue factor are present in atherosclerotic plaque [37]. Tissue factor positive MV and the association of platelet-endothelium and -monocyte interactions with CIMT, support the premise that women with histories of PE have sustained pro-coagulatory and inflammatory vascular compartments, including elevations of hs-CRP (Table 1).

Although the number of women studied in each group was small, a strength of the study is that participants had pregnancy and cardiovascular status confirmed by review of the medical records rather than by self-report. All but one woman were white Caucasian, thus perhaps limiting the generalizability of the results to broader populations. Because this study evaluated women at one point in time many years after their index pregnancies (≈ 35 years), longitudinal studies are needed to better understand temporal/causal patterns of cellular activation for women prior to their pregnancies and as they age past their pregnancies. Some evidence suggests that a subgroup of women may have elevated cardiovascular risk and CIMT prior to their pregnancies [38]. Additional studies are needed to determine what subgroups of women might be predisposed to cardiovascualr risk by pregnancy due to non-conventional risk factors. Such studies would allow development of targeted preventive and therapeutic approaches to reduce risk of PE and overall cardiovascular risk in these women.

In conclusion, activation and interactions among specific intravascular cells are influenced by pregnancy history and could initiate and/or contribute to the progression of subclinical atherosclerosis in postmenopausal women. Therefore, pregnancy history should be accounted for when considering mechanistic analysis and biomarkers idenfication for subclinical atherosclerosis in women.

## Abbreviations

CIMT: carotid intima-media thickness
ICAM-1: intercellular adhesion molecule-1
MV: microvesicles
NP: normotensive pregnancy
PE: preeclampsia
PC: principal component
VCAM-1: vascular cell adhesion molecule-1.
